# Reliability and validity of ultrasonographic automated length measurement system for assessing talofibular anterior instability in acute lateral ankle sprain

**DOI:** 10.1038/s41598-023-30079-z

**Published:** 2023-02-22

**Authors:** Masashi Kawabata, Yusuke Kumazawa, Kazuya Takagi, Hirokazu Okada, Kazuma Miyatake, Takumi Kobayashi, Yuta Nanri, Tomonori Kenmoku, Hiroyuki Watanabe, Naonobu Takahira

**Affiliations:** 1 Department of Rehabilitation, School of Allied Health Sciences, Kitasato University, 1-15-1 Kitazato, Minami-Ku, Sagamihara, Kanagawa 252-0373 Japan; 2 Kumazawa Orthopedic Clinic, Tokyo, Japan; 3 KONICA MINOLTA, INC., Tokyo, Japan; 4 Okada Orthopaedics Clinic, Niigata, Japan; 5 Department of Orthopaedic Surgery, Yokohama City University, Yokohama, Japan; 6 Department of Rehabilitation, Faculty of Health Science, Hokkaido Chitose College of Rehabilitation, Hokkaido, Japan; 7 Department of Rehabilitation, Kitasato University Hospital, Sagamihara, Japan; 8 Department of Orthopaedic Surgery, School of Medicine, Kitasato University, Sagamihara, Japan

**Keywords:** Medical research, Clinical trials

## Abstract

Ankle joint instability after acute lateral ankle sprain (LAS) is an important factor for deciding treatment strategies. Nevertheless, the degree of ankle joint mechanical instability as a criterion for making clinical decisions is unclear. This study examined the reliability and validity of an Automated Length Measurement System (ALMS) in ultrasonography for assessing real-time anterior talofibular distance. Using a phantom model, we tested whether ALMS could detect two points within a landmark following movement of the ultrasonographic probe. Furthermore, we examined whether ALMS was comparable with the manual measurement method for 21 patients with an acute LAS (42 ankles) during the reverse anterior drawer test. Using the phantom model, ALMS measurements showed excellent reliability, with errors below 0.4 mm and with a small variance. The ALMS measurement was comparable to manually measured values (ICC = 0.53–0.71, p < 0.001) and detected differences in talofibular joint distances between unaffected and affected ankles of 1.41 mm (p < 0.001). ALMS shortened the measurement time by one-thirteenth for one sample compared to the manual measurement (p < 0.001). ALMS could be used to standardize and simplify ultrasonographic measurement methods for dynamic joint movements without human error in clinical applications.

## Introduction

A lateral ankle sprain (LAS) is one of the most common ankle injuries, with a recurrence rate above 50%^1,2^. In the United States, an estimated 2 million acute LAS occur each year, resulting in an aggregate health care cost of 2 billion USD^3^. In most cases, LAS heals with conservative treatment, although approximately 20% of cases result in a chronic ankle instability (CAI) subsequent to repeated LAS^3,4^. Dysfunctional outcomes of CAI include a limited range of ankle dorsiflexion, decreased external muscle strength, and static and dynamic postural stability deficits^1,3^. In addition, CAI leads to an increased risk of future ankle osteoarthritis^1,4^.

There is no evidence indicating which type of conservative treatment is more effective for patients with acute LAS^5^. Although the severity of LAS is determined by the magnitude of the ligament rupture and the level of the ankle joint mechanical instability, limit evidence is available that quantifies the degree of the ankle joint mechanical instability for patients with acute LAS^6^. This poses a problem in understanding the severity of LAS, which is central to the clinician’s decision-making process regarding specific rehabilitation protocols and the time needed to return to play^6^. Quantifying the degree of ankle joint mechanical instability in patients with acute LAS could be crucial to the development of a functional diagnosis and enhanced rehabilitation protocols.

Stress ultrasonography (US) has the potential to quantify the magnitude of the ankle instability at the point of care using a radiation‐free and noninvasive approach, as opposed to stress radiography^7–9^. A strong correlation was found between the US and fluoroscopic values measured during simulated anterior drawer and talar tilt tests in a cadaveric ligament transection model^8^. Nevertheless, it remains difficult to quantify the US measurements during dynamic evaluations. In manual anterior drawer testing (ADT), there are technical problems not only associated with the examiner, such as the test skill and the probe operation, but also human-induced measurement errors can occur using electronic calipers or Image J data analysis^10–13^. Even if a bone landmark and/or the attached ligament point were defined, the bone variation or injured deformation may likely be influenced by the bias of the examiner. Moreover, determining the exact timing of the maximum talofibular joint interval during the ADT is difficult, even with an expert examiner. As a result, the human-induced measurement error must be excluded since it is necessary to clarify joint interval changes of less than several millimeters on the US video, even if the examiner’s technical problem has been resolved^14^.

Therefore, we developed the Automated Length Measurement System (ALMS, Japanese Patent Application No. 2022-66,460, KONICA MINOLTA, INC., Tokyo, Japan) to exclude the human measuring errors in US videos. The ALMS can detect the displacement of high-echo areas in real time in the US videos and can judge the maximal-minimal distance between two points. The ALMS may be implemented in clinical practice as a useful functional diagnostic measurement tool if its reliability and validity are proven for patients with acute LAS. This study aimed to examine the reliability of ALMS for US videos and the validity of ALMS for assessing the anterior talofibular distance, compared with the traditional manual measurement method in patients with acute LAS.

## Materials and methods

### Examining ALMS reliability using a phantom model

**Figure 1:**
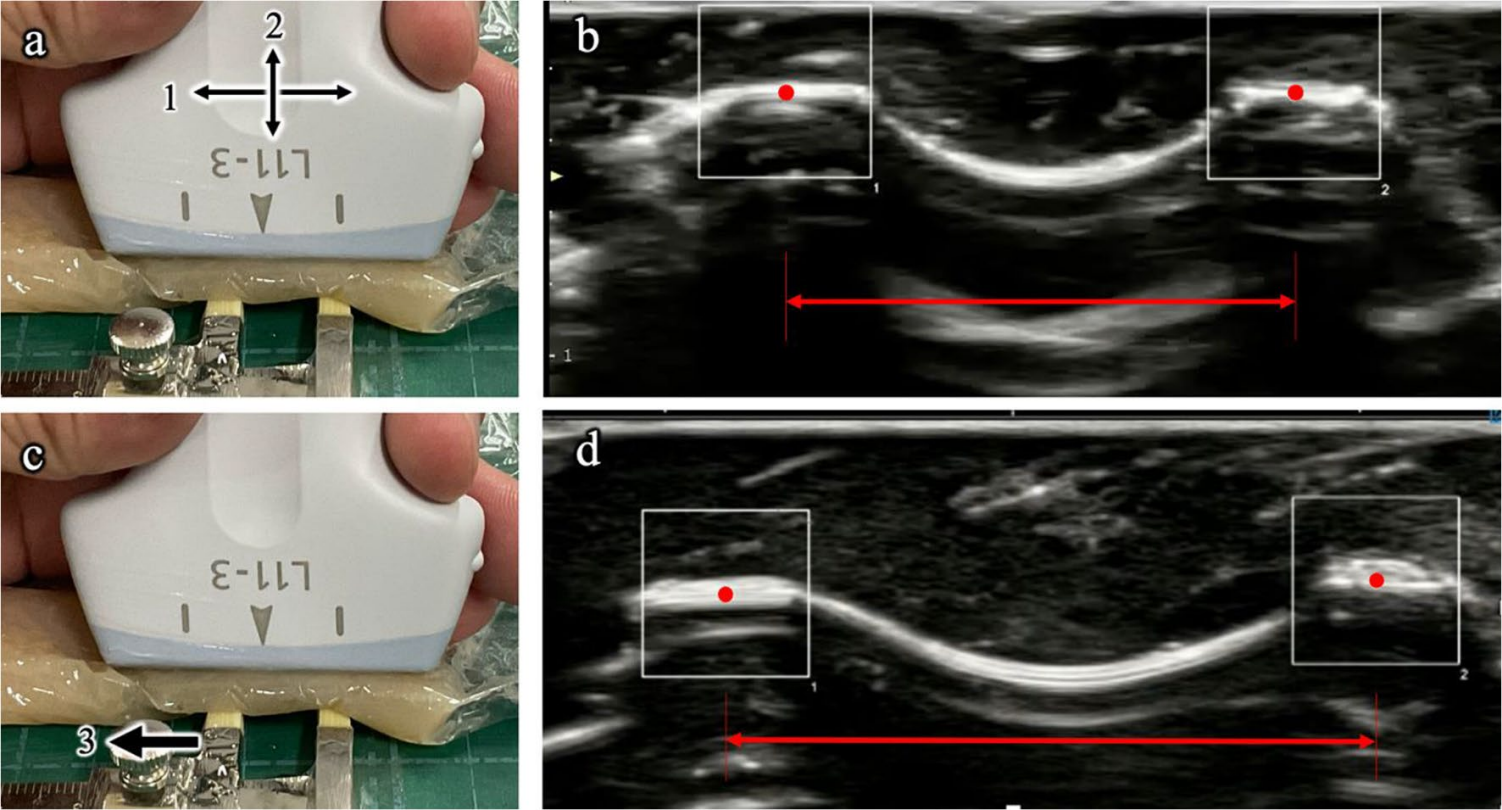
Setup of the reliability examination and ultrasound imaging. (**a**) Lateral (Task 1) and near-far (Task 2) probe movements along the 10-mm caliper distance using a chicken fillet as an intervening substance. (**b**) The red double arrow is the distance between two points of the region of interest (ROI) (small, white circles). (**c**) The caliper is expanded by 5 mm to 15 mm and then manually returned to 10 mm (Task 3). (**d**) The red double arrow is the distance between two points of the ROI, tracking the expansion of the caliper to 15 mm.

We performed pilot experiments using a phantom to examine the reliability of ALMS in ultrasonographic videos. The phantoms, chicken fillets wrapped in a thin plastic film as an intervening substance for the caliper (stainless steel, 0–100 mm), were placed onto the working seat (Fig. 1a,c). The same examiner (M.K, physiotherapist), with over 3 years of experience using musculoskeletal ultrasonography (SONIMAGE MX1 SNiBLE yb, KONICA MINOLTA, INC., Tokyo, Japan), mounted the 11-MHz linear probe onto the phantom. Using a caliper fixed at a distance of 10 mm (true value), the examiner manually moved the ultrasonographic probe in the lateral (Task 1) and near-far (Task 2) directions (Fig. 1a). As the examiner positioned the ultrasonographic probe on the phantom, the caliper distance was manually expanded by the examiner to 15 mm (an increase of 5 mm) and then returned to 10 mm (Task 3, Fig. 1c). These three tasks were performed independently five times by attaching and detaching the probe from the phantom. All simulations were recorded in B-mode; additionally, the video data for the sample were transferred to a PC via an external hard disk drive.

We investigated whether ALMS could follow the probe movement in Tasks 1 and 2 while maintaining the true value of 10 mm and in Task 3, whether it could follow a change in the true value, i.e., an increase of 5 mm. ALMS was activated on the offline PC; then, another examiner (K.T, an engineer) mounted the regions of interest (ROIs) independently on the two top points of the high-echo areas (Fig. 1b,d; small, red circles). These ROIs independently tracked the high-echo area in the videos of Tasks 1–3. The pixel position data of the two ROIs were output at 30 Hz into an Excel file, and the distance between the two ROIs was assessed for reliability using the mean value, standard deviation (SD), standard error of the mean (SEM), and 95% confidence intervals (CIs) of five trials.

### ALMS validity examination in patients with an acute LAS

#### Collection of ultrasonographic data

This study was conducted in accordance with the Declaration of Helsinki and was approved by the Ethics Committee of Kitasato University (study number: 2021-025). The requirement for informed consent was waived by this committee before the start of this retrospective study, given that only anonymized clinical data were used for the analyses conducted in the study.

**Figure 2:**
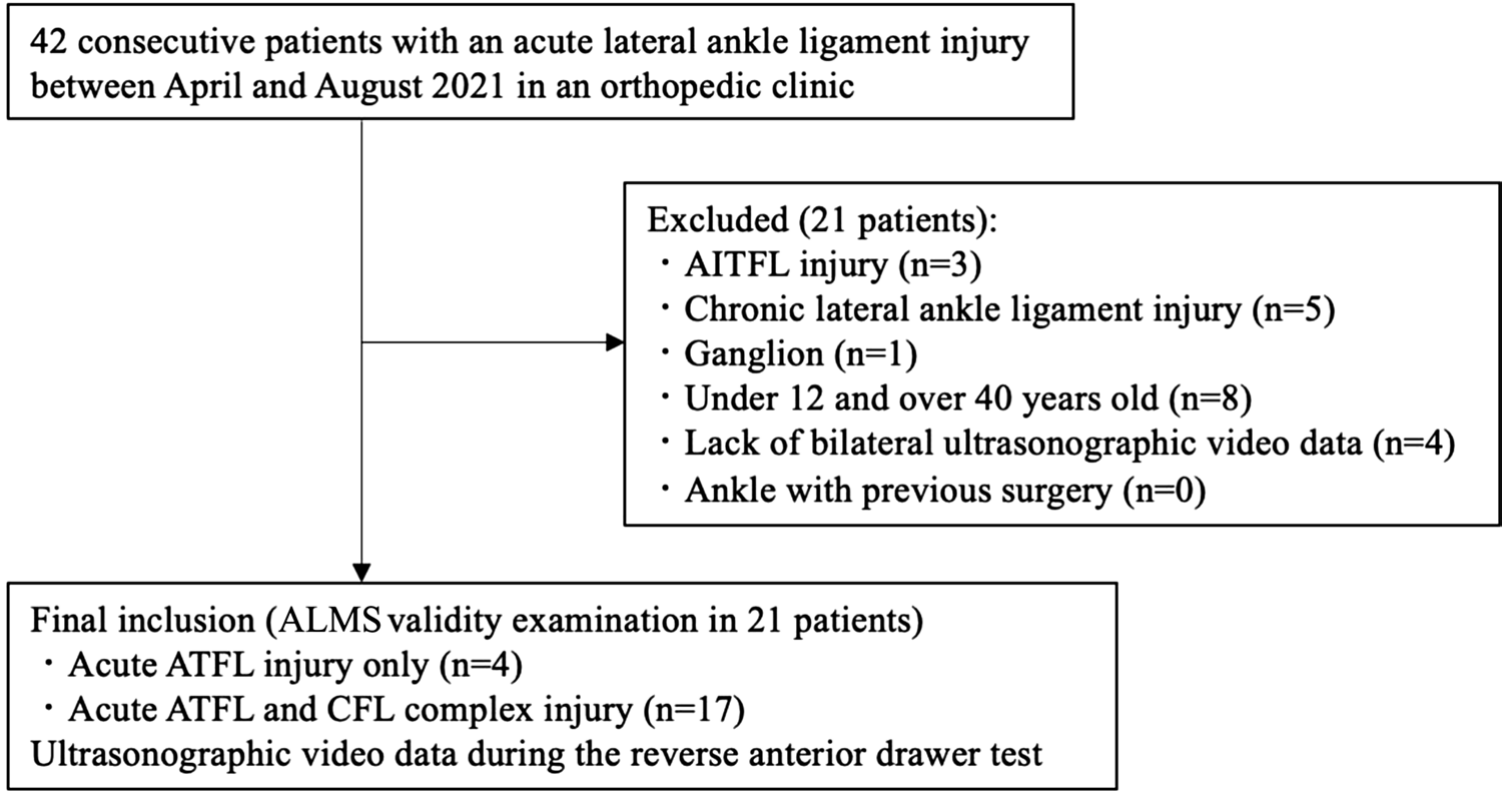
Flowchart of the patient selection process. *AITFL* anterior inferior tibiofibular ligament, *ALMS* Automated Length Measurement System, *ATFL* anterior talofibular ligament, *CFL* calcaneofibular ligament.

In this cross-sectional study, 42 patients with an acute LAS were examined between April and August 2021 in an orthopedic clinic. The inclusion criteria were patients aged over 12 years to under 40 years old with first time event of acute LAS. The exclusion criteria were anterior inferior tibiofibular ligament injury (3 cases), chronic lateral ankle ligament injury (5 cases), ganglion (1 case), age under 12 or over 40 years (8 cases), lack of contralateral ultrasonographic video data (4 cases), and ankle with previous surgery (0 case) (Fig. 2). Lastly, to evaluate the validity of the study, US video data during the reverse anterior drawer test (R-ADT) of 42 ankles of the affected and unaffected side (21 patients with acute LAS) were included (mean age: 17.8 years [standard deviation: 5.5 years, range: 12–34 years]); demographic data are shown in Table 1.

**Table 1:** Demographic data of participants.

	Sample size (%)
Sex (male/female)	12/9
Acute ATFL injury only	4 (19.0%)
Acute ATFL and CFL complex injury	17 (81.0%)
Occurrence	
Basketball	5 (23.8%)
Soccer	4 (19.0%)
Stairs	3 (14.3%)
Baseball	2 (9.5%)
Others	7 (33.3%)

*ATFL* anterior talofibular ligament, *CFL* calcaneofibular ligament.

The sonographer (5 years of experience in musculoskeletal ultrasound) performed all R-ADT, on both affected and unaffected ankles for patients with an acute LAS, during the medical examination within 12 days following the date of the injury. R-ADT was performed while the patient sat on the bed with the knee extended on the chair and the heel in contact with the chair, with approximately 10–15 degrees of plantar flexion^15^. The sonographer placed the transducer (L18-4 Linear Array Transducer, SONIMAGE HS1 SNiBLE, KONICA MINOLTA, INC., Tokyo, Japan) over the origin and insertion points of the long axis of the anterior talofibular ligament (ATFL). The bony landmarks and the anterolateral aspect of the lateral malleolus were identified as the ATFL origin, and the anterolateral aspect of the talus was identified as the insertion point^14^. These bony landmarks can be identified by their hyperechogenic area, and the transducer was fine-tuned during the ankle movement to continue to describe the clear bony outline and the long axis of ATFL.

**Figure 3:**
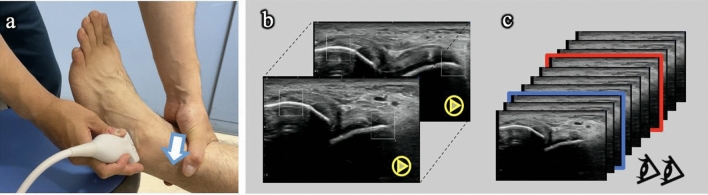
Sonographic data collection and measurement methods. (**a**) Reverse anterior drawer test from non-stress to loading conditions of the tibia posterior (arrow), viewed along the ATFL long axis. (**b**) ALMS; Sonographic video with one amplitude of joint motion cut/selected offline, followed by attachment of the regions of interest (ROI)s to two points on top of the talofibular joint to detect the trajectory. (**c**) Manual measurement; The two testers measure the joint distance individually by converting static images and judging minimal (blue) and maximal (red) distances. *ALMS* automated length measurement system, *ATFL* anterior talofibular ligament.

With one hand, the sonographer held the distal tibia while pushing the base of the palm against it to induce a posterior displacement of the tibia parallel to the articular surface of the talus by applying the ground reaction force to the heel (Fig. 3a). The reverse anterior lateral drawer test involved applying a ground reaction force to the heel and was more sensitive and accurate (sensitivity and specificity were 0.92 and 0.88 for the senior examiner, and 0.87 and 0.91 for the junior examiner, respectively) than the ADT using only manual anterior drawer stress (0.40 and 0.50 for the senior examiner, and 0.05 and 0.48 for the junior examiner, respectively) in diagnosing chronic ATFL injuries^16^. All procedures were recorded in B-mode; additionally, the video sample data were transferred to a PC via an external hard disk drive.

#### ALMS and manual measurements

All raw ultrasonographic video data were trimmed to only one amplitude of joint translation during R-ADT (maximum– minimum joint distance) using Final Cut Pro (Apple. Inc.). Using these trimmed videos, the examiner (K.T, engineer) mounted the ALMS of the ROI on the two top points of the high-echo area in the talofibular joint (Fig. 3b). The minimum and maximum changes in the location and distance between two ROIs were calculated after setting the output to 30 Hz in the Excel file. In addition, the measurement time required per video sample was calculated from establishing the ROIs to exporting the finished results to Excel.

Next, the same trimmed videos were converted into several consecutive static images at 10 Hz with a VLC media player (Fig. 3c). Two independent testers (physiotherapy students) manually measured the joint distance between the talofibular joint base at the ATFL origin and insertion points on several images individually using Image J (NIH, Maryland, USA). These students were repeatedly instructed by M.K. and were well trained in determining variation in the bone form and portion of ligament insertion.

A comparison was made between the manual and ALMS measurement methods for the evaluation of the performance validity of the ALMS using changes in the joint distance based on the minimum and maximum distance between two ROIs. In addition, the measurement time required for one series of image samples was measured using Image J, and the finished output was exported to Excel.

#### Statistical analyses

The interobserver reliability between the ALMS and manually measuring testers was evaluated by using the intraclass correlation coefficient (ICC) with a two-way random effects model, assuming a single measurement and absolute agreement. ICC values were interpreted as follows: poor agreement, < 0.5; moderate agreement, 0.5–0.74; good agreement, 0.75–0.9; and excellent agreement, > 0.9^17^.

We compared the mean difference of the talofibular joint distance between the ALMS and two manual testers using one-way analysis of variance (ANOVA) followed by Bonferroni’s multiple comparison test. Using paired *t* tests, we compared the mean difference between the unaffected and affected talofibular joint distances during R-ADT measurement methods. In addition, the measurement time required per sample between ALMS and manual measurement methods was also assessed using the paired *t *test. SPSS Statistics version 22.0 (IBM Corp, New York, USA) was used to perform all statistical analyses, and p < 0.05 was considered statistically significant.

The sample size was calculated by an a priori power analysis using the G*Power (version 3.1). Based on an α of 0.05, a power of 0.80, and an effect size of 0.46 and with respect to the results of a previous sonography study^6^, size analysis indicated that a total sample size of 39 would be required to demonstrate a significant difference in the paired *t* test.

## Results

### ALMS reliability examination using a phantom model

Results of ALMS reliability tests are shown in Table 2. The ALMS was able to track the distance of the caliper (10 mm) within an error range of < 0.4 mm and with a small variance (SEM) in Tasks 1 and 2 repeated five times. In addition, the ALMS was able to track the 15-mm expanded distance of the caliper within an error range of < 0.3 mm and with a small variance in Task 3 (Supplementary Fig. 1).

**Table 2:** Results of the reliability examination, repeated five times.

		Mean true value	Mean max–min	SD	SEM	95% CI
Task 1. Probe lateral movement	mm	10.34	0.30	0.12	0.06	0.18–0.41
Task 2. Probe near-far movement	mm	10.39	0.38	0.05	0.02	0.33–0.42
Task 3. Expansion of the caliper	mm	–	4.70	0.33	0.15	4.38–5.01

*max* maximal, *min* minimal, *SD* standard deviation, *SEM* standard error of the mean, *CI* confidence interval.

### ALMS validity assessment in patients with an acute LAS

The interobserver reliability between ALMS and the manually measuring testers had a moderate-to-good agreement (ICC_2.1_ = 0.53–0.81) (Table 3). There was no significant difference in the absolute value (maximal and minimal distance) using the three measurement methods. Conversely, there were significant differences in terms of relative value (difference maximal-minimal and ratio) in only the unaffected side, in particular between the ALMS and the manual tester A (Table 4). Changes in the talofibular joint distance in the affected and unaffected ankles during R-ADT are shown in Table 5. Differences in the maximal to minimal distance between the affected and unaffected ankles were 1.33–1.47 mm in three measurement methods. All parameters, except for minimal distance, were significantly different between the affected and unaffected ankles for the ALMS and two manual testers (Table 5). Compared to the manual method (mean 104.6 s, SD 21.7 s), the mean measurement time for one sample was significantly shorter with the ALMS (mean 8.2 s, SD 3.6 s) (p < 0.001, 95% CI 90.3–102.5).

**Table 3:** Inter-rater reliability of ALMS and manual measurements for the talofibular joint distance between the ATFL origin and insertion points.

	ALMS vs. manual tester A			ALMS vs. manual tester B			Manual tester A vs. manual tester B		
	ICC	95% CI	p-value	ICC	95% CI	p-value	ICC	95% CI	p-value
Minimal distance	0.63	0.41–0.79	0.001	0.62	0.38–0.77	0.001	0.81	0.68–0.89	0.001
Maximal distance	0.63	0.41–0.78	0.001	0.71	0.56–0.83	0.001	0.80	0.64–0.89	0.001
Difference (max–min)	0.53	0.07–0.77	0.001	0.59	0.32–0.77	0.001	0.67	0.43–0.81	0.001

*ATFL* anterior talofibular ligament, *max* maximal, *min* minimal, *ALMS* automated length measurement system, *ICC* intraclass correlation coefficient, *CI* confidence interval.

**Table 4:** Comparison of the mean difference of the talofibular joint distance between the ATFL origin and insertion points during the reverse anterior drawer test between the ALMS and two manual testers.

		ALMS	Manual tester A	Manual tester B	F (2,60)	p-value
		Mean ± SD	Mean ± SD	Mean ± SD		
Unaffected side						
Minimal distance	mm	22.59 ± 3.48	21.34 ± 3.14	21.29 ± 2.96	1.12	0.333
Maximal distance	mm	23.48 ± 3.58	23.14 ± 3.23	22.72 ± 2.97	0.29	0.751
Difference (max–min)	mm	0.89 ± 0.60^#^	1.80 ± 0.92	1.43 ± 0.85	6.93	0.002*
Ratio (max/min)		1.04 ± 0.03^#^	1.09 ± 0.06	1.07 ± 0.05	5.26	0.008*
Affected side						
Minimal distance	mm	22.84 ± 2.61	22.60 ± 2.20	22.27 ± 2.34	0.31	0.737
Maximal distance	mm	25.15 ± 2.91	25.87 ± 2.82	25.03 ± 2.53	0.57	0.567
Difference (max–min)	mm	2.30 ± 1.43	3.27 ± 1.39	2.76 ± 1.01	2.94	0.061
Ratio (max/min)		1.10 ± 0.07	1.14 ± 0.06	1.13 ± 0.05	2.54	0.088

*ATFL* anterior talofibular ligament, *ALMS* automated length measurement system, *max* maximal, *min* minimal, *SD* standard deviation.

*Significant difference of talofibular joint distance at p < 0.05 (one-way ANOVA).

^#^Significant difference between ALMS and Manual tester A at p < 0.05 (Bonferroni’s multiple comparison test).

**Table 5:** Comparison of differences between affected and unaffected ankles in talofibular joint distance between the ATFLs during the reverse anterior drawer test.

		Difference between affected and unaffected side		p-value
		Mean	95% CI	
ALMS				
Minimal distance	mm	0.25	− 1.16–1.66	0.718
Maximal distance	mm	1.66	0.19–3.14	0.029*
Difference (max–min)	mm	1.41	0.71–2.12	0.001*
Ratio (max/min)		0.06	0.03–0.10	0.001*
Manual tester A				
Minimal distance	mm	1.26	− 0.13–2.65	0.073
Maximal distance	mm	2.73	0.87–4.59	0.006*
Difference (max–min)	mm	1.47	0.59–2.35	0.002*
Ratio (max/min)		0.06	0.02–0.10	0.009*
Manual tester B				
Minimal distance	mm	0.98	− 0.63–2.58	0.217
Maximal distance	mm	2.31	0.77–3.84	0.005*
Difference (max–min)	mm	1.33	0.61–2.04	0.001*
Ratio (max/min)		0.06	0.01–0.10	0.010*

*ATFL* anterior talofibular ligament, *ALMS* automated length measurement system, *max* maximal, *min* minimal, CI confidence interval.

*p < 0.05 (paired *t* tests).

## Discussion

Herein, we present novel findings evaluating the applicability of the ALMS as a highly reliable and moderately valid method for tracking movable objects in an ultrasonographic video. The ALMS was able to detect a moving phantom object with an error of less than 0.4 mm with a small variance. Furthermore, the ALMS required a remarkably short measurement time and exhibited moderate validity compared with the manual measurement method for patients with an acute LAS during R-ADT. The ALMS could be clinically applied as a standard to simplify the ultrasonographic measurement method without human-induced errors for dynamic joint movements.

### Evaluation of ALMS reliability

The ALMS was able to track the true value of the movable phantom distance with an error of less than 0.4 mm and with a small variance (average error of approximately 3–4% against the true value). The results have significant implications for ultrasonography since it is always challenging to quantify a change in millimeters without an error. In particular, results from the lateral (Task 1) and near-far (Task 2) probe movements showed that human-induced probe movement is possible. Using ultrasonography, the examiner needs to make a fine adjustment to the probe for the talofibular joint to clearly draw the long axis of the ATFL fiber and the contour of the bone. In addition, the results of the expanded distance of the phantom (Task 3) demonstrated that the true value could be tracked in real time as it changed. ALMS measurements of the two ROIs must independently tracked the change in the two high-echo areas in real time. A significant ability to track changes in the target object would be required to calculate the minimum and maximum distance of the talofibular joint. The most important issue in an ankle stress test is to reproducibly judge the precise moment of the minimal or maximal distances in real time, even when performed by an expert. The ALMS could detect change in two high-echo areas in real time and represents a momentous measurement system devoid of ultrasonographic human-induced measurement errors.

### ALMS validity examination for patients with an acute LAS

Compared with manual measurements, the ALMS performed moderately well (ICC_2.1_ = 0.53–0.71) in assessing patients with an acute LAS. The two manual inter-testers were comparable, with a moderate-to-good agreement (ICC_2.1_ = 0.67–0.81). In addition, although absolute value (maximal and minimal distance) had no significant difference in three measurement methods, the relative value (difference between the maximal-minimal values and ratios) in the unaffected side showed significant differences. These results indicate that perfect matching was difficult for the manual testers, even if the same series of static images unified the landmark. There may be subtle differences in the landmarks that are affected by the ATFL thickness or the bone shape variance in manual measurements. Because assessors may have individual priorities and visual performances, the gold standard for automated length measurement system must exclude human-induced variability. The ALMS, which exhibited good validity for manual variability, can contribute to unifying measurements.

Based on the magnitude of the ankle instability, the change in the joint distance from maximal to minimal values during R-ADT between the unaffected and affected ankles was reasonable for the ALMS (1.41 mm) and the two manual measurements (1.33 and 1.47 mm). The results of our study support several previous studies reporting that the change in the joint distance during ADT was less than 1.0 mm (< 1.10 ratio) in a healthy population^7,15^. Song et al. reported that the mean uninjured ATFL length was 19.2 mm (16.5–21.0 mm) at rest and 20.2 mm (17.8–21.3 mm) under stress during ADT; thus, the difference between resting and stress was 1.0 mm (0.0–3.1 mm)^15^. Yokoe et al. reported that the ATFL length ratio was 1.08–1.09 during ADT in healthy Japanese participants^7^. There are, however, few similar studies that compare acute and CAI measurements. For CAI, the magnitude of the anterior translation measured by stress radiography using the Telos stress device was inconsistent, e.g. either more than 5 mm^14^ or 3.2–21.0 mm^18^. Separated by more or less than 5 mm using stress radiography, the mean ATFL lengths under stress ultrasonography during manual ADT were 1.9 mm and 2.2 mm for patients with < 5 mm and > 5 mm translation, respectively^14^. Mizrahi et al. also reported that the mean ATFL length difference (neutral and stressed) in ultrasonography was 1.26 mm (17.22–18.48 mm) in the symptomatic population compared with 0.44 mm (18.12–18.56 mm) in the asymptomatic population^12^.

Although index measures of CAI have often been determined used the Telos stress device, it is necessary to develop a different index for use with US to determine acute ankle instability. As a means of diagnosing acute ATFL injuries and painful chronic ankle instabilities, the US is characterized by greater advantages than stress radiography, as the endpoint of the stretched ATFL can be visualized, and there is no need for inducing such a high magnitude of stress using the Telos device.

Compared to manual measurement, the ALMS shortened the measurement time in our prototype by 1/13th for one sample. In many cases, the distance was measured and considerable time was spent using electronic calipers or Image J^10–13^. Thus, the ALMS can find practically application for assessing an acute LAS in a clinical setting and may serve as a gold standard.

There were several limitations to this study. First, this was a preliminary study; hence, further samples are needed to analyze the cutoff value for judging joint instability. Second, for the functional assessment, it is necessary to examine changes in instability on ligament recovery. Lastly, a prospective study is needed to examine the relationship between the ankle joint function and a return to sports activity.

In conclusion, the ultrasonographic ALMS was able to accurately detect the phantom distance change between two high-echo areas of an assumed joint movement. Furthermore, the validity of the ALMS was confirmed, compared with manual measurement methods, for assessing the anterior talofibular distance in patients with an acute LAS. ALMS could be a momentous measurement system for clinical application, which eliminates human error from ultrasonographic measurements.

## Supplementary Information

Supplementary Figure 1.

## Data Availability

The data presented in this study are available on request from the corresponding author.

## References

[1] Kobayashi T (2014). The effects of a semi-rigid brace or taping on talocrural and subtalar kinematics in chronic ankle instability. Foot Ankle Spec..

[2] Kobayashi T, Koshino Y, Miki T (2021). Abnormalities of foot and ankle alignment in individuals with chronic ankle instability: A systematic review. BMC Musculoskelet. Disord..

[3] van Rijn RM (2008). What is the clinical course of acute ankle sprains? A systematic literature review. Am. J. Med..

[4] Lee S, Song K, Lee SY (2022). Epidemiological study of post-traumatic ankle osteoarthritis after ankle sprain in 195,393 individuals over middle age using the National Health Insurance Database: A retrospective design. J. Sci. Med. Sport.

[5] Ortega-Avila AB (2020). Conservative treatment for acute ankle sprain: A systematic review. J. Clin. Med..

[6] Wisthoff BA (2021). Identifying range-of-motion deficits and talocrural joint laxity after an acute lateral ankle sprain. J. Athl. Train..

[7] Yokoe T (2021). The ratio of stress to nonstress anterior talofibular ligament length on ultrasonography: Normative values. Orthop. J. Sports Med..

[8] Saengsin J (2022). Use of portable ultrasonography for the diagnosis of lateral ankle instability. J. Orthop. Res..

[9] Cho JH (2016). Value of stress ultrasound for the diagnosis of chronic ankle instability compared to manual anterior drawer test, stress radiography, magnetic resonance imaging, and arthroscopy. Knee Surg. Sports Traumatol. Arthrosc..

[10] Tsutsumi K (2022). Feasibility of an ultrasound-based method for measuring talar displacement during the anterior drawer stress test using a telos device: A preliminary study. Int. J. Environ. Res. Public Health.

[11] Kikumoto T (2019). Quantitative evaluation method for clarifying ankle plantar flexion angles using anterior drawer and inversion stress tests: A cross-sectional study. J. Foot Ankle Res..

[12] Mizrahi DJ, Nazarian LN, Parker L (2018). Evaluation of the anterior talofibular ligament via stress sonography in asymptomatic and symptomatic populations. J. Ultrasound Med..

[13] Matsuo K (2022). Medial elbow joint space gapping associated with repetitive baseball pitching in preadolescent baseball players. J. Shoulder Elbow Surg..

[14] Lee KT (2014). New method of diagnosis for chronic ankle instability: Comparison of manual anterior drawer test, stress radiography and stress ultrasound. Knee Surg. Sports Traumatol. Arthrosc..

[15] Song JH (2021). Evaluation of the uninjured anterior talofibular ligament by ultrasound for assessing generalized joint hypermobility. Foot Ankle Surg..

[16] Li Q (2020). Reverse anterolateral drawer test is more sensitive and accurate for diagnosing chronic anterior talofibular ligament injury. Knee Surg. Sports Traumatol. Arthrosc..

[17] Choi JH (2021). Consistency and reliability of ankle stress radiography in patients with chronic lateral ankle instability. Orthop. J. Sports Med..

[18] Guerra-Pinto F (2021). Lack of definition of chronic ankle instability with arthrometer-assisted ankle joint stress testing: A systematic review of in vivo studies. J. Foot Ankle Surg..

