# Supplementary material for: Reliability and validity of ultrasonographic automated length measurement system for assessing talofibular anterior instability in acute lateral ankle sprain
Source: Sci Rep. 2023 Feb 22;13:3098. doi: 10.1038/s41598-023-30079-z (PMC9947169; doi:10.1038/s41598-023-30079-z)
Supplement: Supplementary file 1 — Supplementary Figure 1. [file 41598_2023_30079_MOESM1_ESM.docx]

**
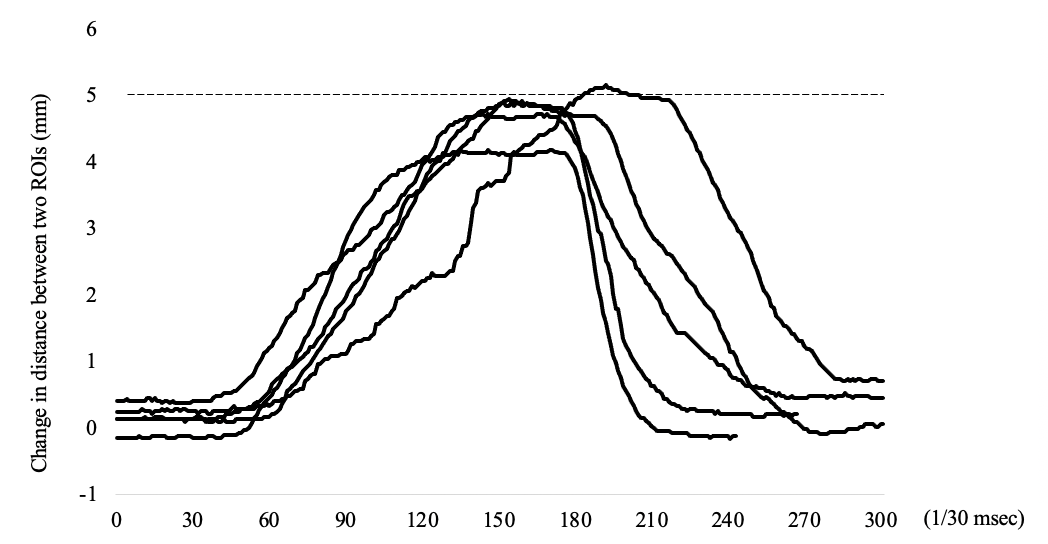
**

**Supplementary Fig 1.** Changes in the distance between two points of the region of interest (ROI) during caliper movements from 10 mm to 15 mm, repeated 5 times.
